# A Combined Molecular Docking/Dynamics Approach to Probe the Binding Mode of Cancer Drugs with Cytochrome P450 3A4

**DOI:** 10.3390/molecules200814915

**Published:** 2015-08-14

**Authors:** Suresh Panneerselvam, Dhanusha Yesudhas, Prasannavenkatesh Durai, Muhammad Ayaz Anwar, Vijayakumar Gosu, Sangdun Choi

**Affiliations:** Department of Molecular Science and Technology, Ajou University, Suwon 443-749, Korea; E-Mails: suresh@ajou.ac.kr (S.P.); dhanusha@ajou.ac.kr (D.Y.); prasanna@ajou.ac.kr (P.D.); ayaz@ajou.ac.kr (M.A.A.); gosu@ajou.ac.kr (V.G.)

**Keywords:** CYP450, drug metabolism, docking, molecular dynamics simulation

## Abstract

Cytarabine, daunorubicin, doxorubicin and vincristine are clinically used for combinatorial therapies of cancers in different combinations. However, the knowledge about the interaction of these drugs with the metabolizing enzyme cytochrome P450 is limited. Therefore, we utilized computational methods to predict and assess the drug-binding modes. In this study, we performed docking, MD simulations and free energy landscape analysis to understand the drug-enzyme interactions, protein domain motions and the most populated free energy minimum conformations of the docked protein-drug complexes, respectively. The outcome of docking and MD simulations predicted the productive, as well as the non-productive binding modes of the selected drugs. Based on these interaction studies, we observed that S119, R212 and R372 are the major drug-binding residues in CYP3A4. The molecular mechanics Poisson–Boltzmann surface area analysis revealed the dominance of hydrophobic forces in the CYP3A4-drug association. Further analyses predicted the residues that may contain favorable drug-specific interactions. The probable binding modes of the cancer drugs from this study may extend the knowledge of the protein-drug interaction and pave the way to design analogs with reduced toxicity. In addition, they also provide valuable insights into the metabolism of the cancer drugs.

## 1. Introduction

Cytochrome P450 (CYPs) contributes to vital life processes by oxidizing compounds, such as drugs, chemicals, pollutants and xenobiotics [1,2]. CYPs are major drug-metabolizing enzymes that play an important role in the oxidative metabolism of the predominantly-used clinical drugs. Among the CYPs isoforms, the CYP3A4 enzyme metabolizes approximately half of the drugs. CYP3A4 has a high level of expression in the liver and broad capacity to oxidize structurally-diverse substrates due to its large active site cavity [3,4]. CYP3A4 has diverse substrate specificity and co-operative substrate binding, which often leads to unacceptable drug-drug interactions, as well as toxic side effects [5]. CYP3A4 can lower the bioavailability and therapeutic efficiency of pharmaceuticals through fast degradation, whereas drug plasma levels can be increased if CYP3A4 is inhibited [6].

Surgery and radiation are used to treat cancers that are locally confined, whereas drug therapy is essential for killing metastatic cancer cells [5]. Cytarabine, daunorubicin, doxorubicin and vincristine are clinically used in different combinations for combinatorial therapies of cancers, such as breast cancer, leukemia, non-Hodgkin lymphoma and acute myeloid leukemia [7,8,9]. Knowledge of how these drugs are metabolized by CYPs is crucial for predicting their bioactivity and toxicity. Moreover, the mechanisms of the toxicity of these drugs are not clear because there are multiple factors that contribute to their toxicity [5]. As far as we are aware, neither metabolic products nor molecular mechanisms of cytarabine, daunorubicin or doxorubicin by CYPs have been elucidated. However, there is a metabolic study on vincristine that proposes the biotransformation of this drug [10].

Determination of the CYP3A4 crystal structures has provided a solid ground for many computational studies that provided insight into the catalytic mechanism and has facilitated a more accurate prediction of small-molecule association with CYP3A4 [6]. Few other computational studies related to CYP3A4 have also been published [11,12,13,14,15] and have been recently reviewed [6]. Understanding the binding conformation of these drugs with CYP3A4 is important, and these models could explain its biotransformation.

Computational advances have led to the development of increasingly successful molecular simulations of protein structural dynamics that are fundamental to biological processes [16,17]. These simulations facilitated the development of models that are consistent with experiments and have provided insights into the associated biological mechanisms [18]. In this study, we used computational molecular docking and molecular dynamics (MD) simulations along with the site of metabolism (SOM) using SMARTCyp [19] to understand the interaction of selected drugs with CYP3A4. This understanding may extend the therapeutic options, reduce the undesirable side effects and will help in computer-aided drug design.

## 2. Results and Discussion

### 2.1. Drug Interaction with CYP3A4

The metabolic action of anticancer drugs cytarabine, daunorubicin and doxorubicin by CYP3A4 is not completely understood to date, whereas the metabolic products have been reported for vincristine [10]. However, it has been reported that these drugs are substrates of CYP3A4 [20]. The detailed binding orientation, as well as the interaction profiles would be essential in understanding the metabolism of these drugs. We have combined docking, MD simulations and SOM using SMARTCyp to predict binding modes. SMARTCyp is a method to predict the SOM in a molecule that is most likely to be metabolized by CYP3A4 and other CYPs isoforms.

**Figure 1:**
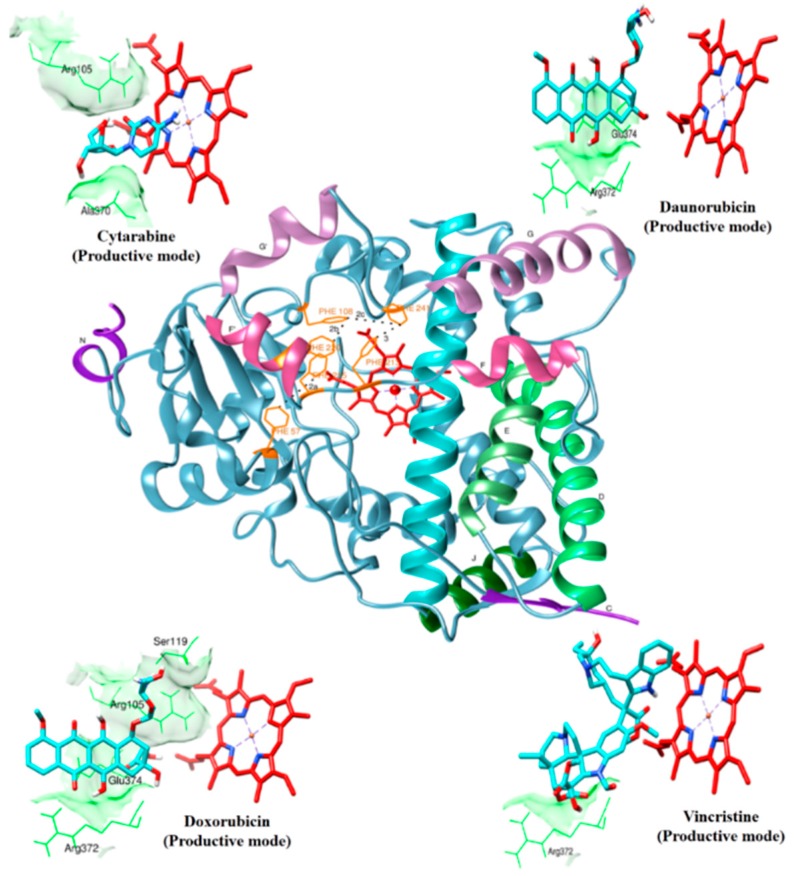
Productive binding modes of cytarabine, daunorubicin, doxorubicin and vincristine docked with CYP3A4 are shown. The overall fold of CYP3A4 is shown in the center. Only the heme (red color in stick representation), drugs (cyan color in stick representation) and hydrogen bonding residues (green color in line representation) are shown. A few hydrogen bonding residues are not shown for clarity.

The productive drug-binding poses (Figure 1) were chosen based on four criteria: (1) docking solutions with a distance less than 0.6 nm between any neighboring atom of drug and the heme iron; (2) the predicted SOM of the drug molecule is oriented towards the heme; (3) consistent docking pose with two different structures of CYP3A4 (1TQN and 4I3Q) in docking; and (4) the stability of the CYP3A4-drug complexes in 50-ns MD simulations. The non-productive binding poses were selected based on three criteria: (1) the docked drugs were within the active site of CYP3A4, but SOM was oriented away from heme; (2) consistent docking poses in the two different CYP3A4 structures used, and (3) the interacting residues agree with the experimentally-proven ones (only applicable for peripheral binding poses). The results for the docked drug molecules are concisely given in Table 1, and the binding modes of those drugs are discussed below in detail. Additionally, a flexible docking approach was applied in the Molecular Operating Environment (MOE; Chemical Computing Group Inc., Montreal, Canada) software to explore the other possible binding modes.

**Table 1:** Summary of molecular interactions of the drugs with CYP3A4.

Drug	Binding Pose	Conformational Clusters Out of 100 Runs	H-Bond Interactions (nm)	Binding Energy (kcal/mol)	Distance between Heme and Ligand (nm)	Van der Waals Interacting Residues
Cytarabine	Productive binding pose		(R105)N-H…O=0.211			R105, R212, A305, T309, A370
		16	(A305)O-H…N=0.193	−5.54	0.223	
			(A370)O-H…O=0.194			
	Non-productive binding pose		(R212)N-H…O=0.193			R212, F215, S312, I369, A370, M371, L382, G481, L483
		20	(L483)O-H…N=0.184	−6.17	0.72	
			(L483)N-H…N=0.220			
Daunorubicin	Productive binding pose		(R372)N-H…O=0.215			F57, R105, R106, F108, S119, F213, F304, R372, E374, heme
			(E374)N-H…O=0.216	−11.81	0.297	
		68	(E374)O…H-O=0.220			
	Non-productive binding pose I		(T224)O…H-N=0.108			F108, F215, F304, S119, R105, F57, R372, E374, L373, A370, F304, heme
			(R372)N-H…O=0.194	−11.69	0.483	
		10	(E374)O…H-O=0.177			
	Non-productive binding pose II	4	(N420)N-H…O=0.273	−	0.69	Y99, W126, R128, R130, R375, P439, N441, I443, F435
Doxorubicin	Productive binding pose		(R105)N-H…O=0.225			F57, D76, R105, R106, S119, F213, F215, F304, A370, R372, E374, R375, heme
			(S119)O…O-H=0.222			
		69	(R372)O…O-H=0.182	−12.17	0.208	
			(E374)N-H…O=0.132			
			(Heme)O…H-O=0.190			
	Non-productive binding pose		(E374)O…H-O=0.202			R105, R106, F108, S119, R212, F215, I301, A305, A370, R372, E374, heme
		15	(E374)O…H-N=0.203	−10.24	0.275	
Vincristine	Productive binding pose		(R372)N-H…O=0.222			F57, R105, F108, S119, I120, R212, F304, A305, F213, F215, M371, R372, G481, L482, heme
		12		−4.42	0.272	
	Non-productive binding pose	2	-	+53.43	0.845	L351, D357, N361, K424, I427, P429, Y432, P434, F435, G436, S437, M445, R446, L449, K453

### 2.2. Cytarabine

Cytarabine is an anticancer chemotherapy drug that has been reported to be metabolized by human CYP3A4, and since it has a cytosine base with an arabinose sugar moiety, it is also called cytosine arabinoside [20]. The docked cytarabine had two major clusters of two different binding modes, each with 16 and 20 binding conformations, respectively, whereas, many minor clusters were also observed in the docked poses that were not perused, owing to selection criteria. Out of these binding modes, one productive and one non-productive binding mode were studied. Other possible binding poses were generated through flexible docking that yielded similar poses to that of rigid docking.

#### 2.2.1. Productive Binding Mode of Cytarabine

**Figure 2:**
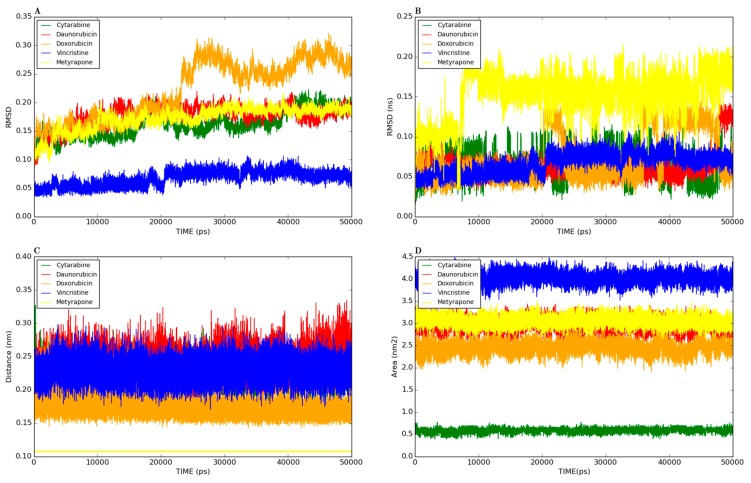
Analyses of the productive binding poses of cytarabine, daunorubicin, doxorubicin, vincristine, cytochrome (alone) and metyrapone (control) were subjected to 50-ns simulations. (**A**) Root mean square deviation (RMSD) of the protein backbone atoms, with respect to the initial structure; (**B**) drug RMSD, with respect to the initial structure; (**C**) The solvent accessible surface area for the drug complexes; (**D**) heme to drug distances measured.

**Figure 3:**
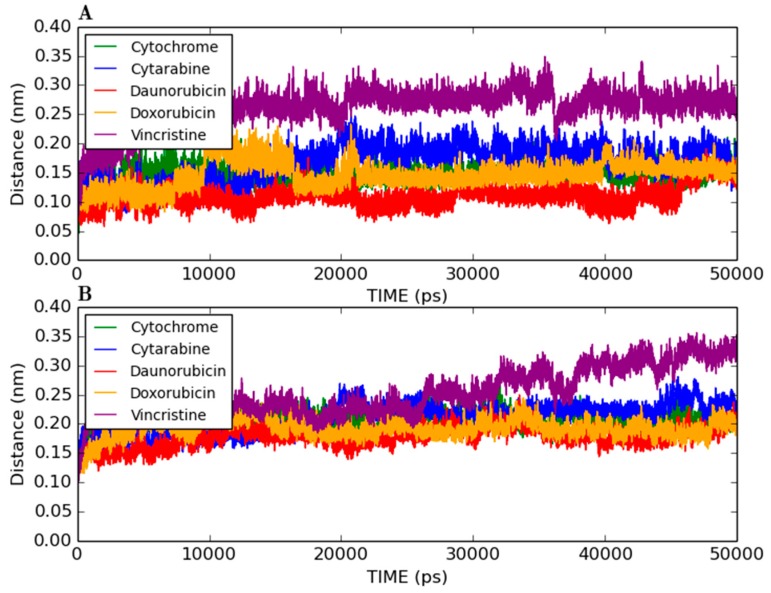
MD analysis of loops. (**A**) RMSD of the phenylalanine loop (F108, F219, F220, F241 and F304); and (**B**) F–G connecting loop (209–217) for the productive binding pose of cytarabine, daunorubicin, doxorubicin and vincristine.

**Figure 4:**
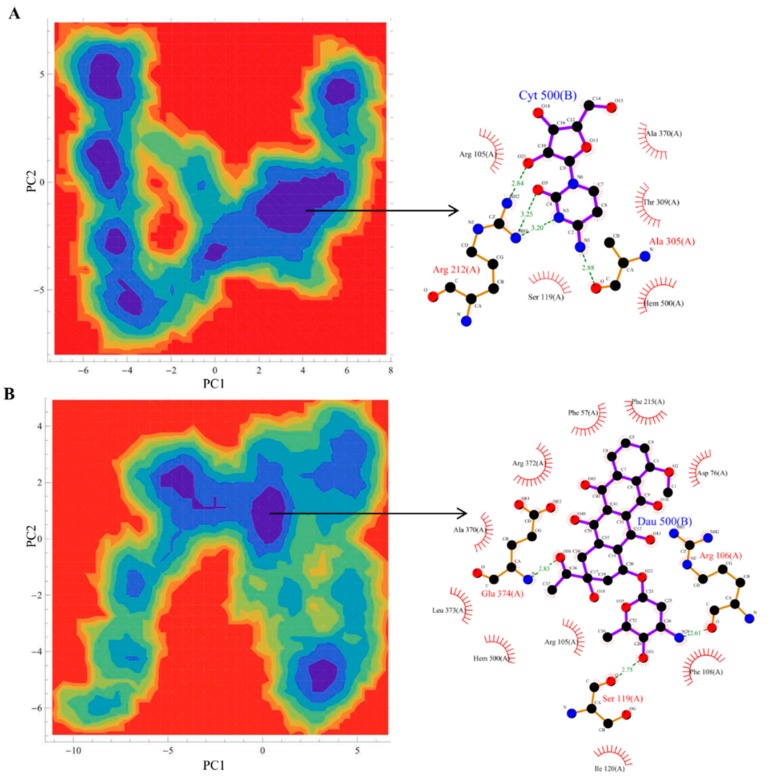
Free energy landscape (FEL) and the representative lowest energy conformation of simulated drugs. The FEL of the simulated complexes based on the principal component analysis: (**A**) cytarabine; (**B**) daunorubicin. Each representative structure is displayed from the most populated free energy minimum clusters using PDBsum.

**Figure 5:**
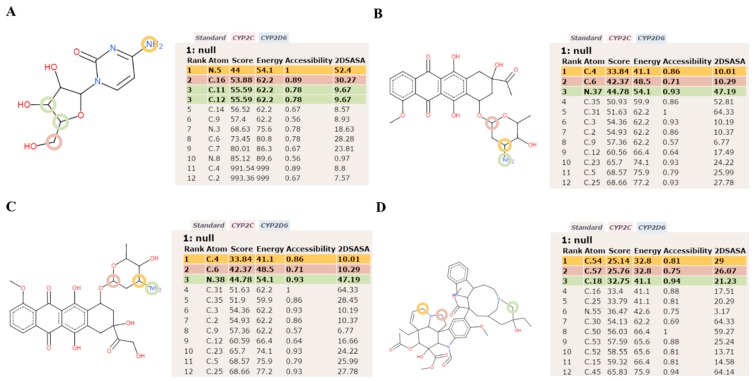
Metabolic site prediction using SMARTCyp for the anticancer drugs. (**A**) The cytarabine structure contains a cytosine base with an arabinose sugar moiety. The primary site of metabolism (SOM) in cytarabine is the amino group in the cytosine base. The secondary predicted SOM is the acetyl carbon (C16), and the tertiary SOM is the 11th carbon in the arabinose sugar moiety; (**B**) Daunorubicin features a tetracyclic ring structure and daunosamine moiety. The primary SOM is a carbon (C4) in the amino sugar. The secondary and tertiary SOMs are carbon (C6) and an amino group of the daunosamine moiety, respectively; (**C**) Doxorubicin has a tetracyclic ring structure and daunosamine moiety. The predicted results of SMARTCyp were similar as those for doxorubicin; (**D**) SMARTCyp predicted the primary SOM as carbon (C54) near the pyrimidine ring out of the two multi-ringed units, vindoline and catharanthine. The secondary SOM is carbon (C57) in the pyrimidine ring of the vindoline ring, and the tertiary SOM is the carbon (C18) in the catharanthine ring of vincristine.

**Figure 6:**
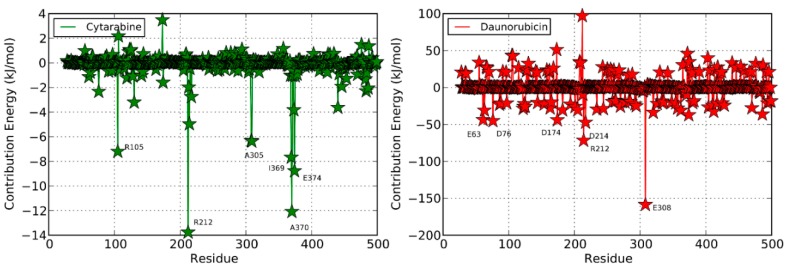

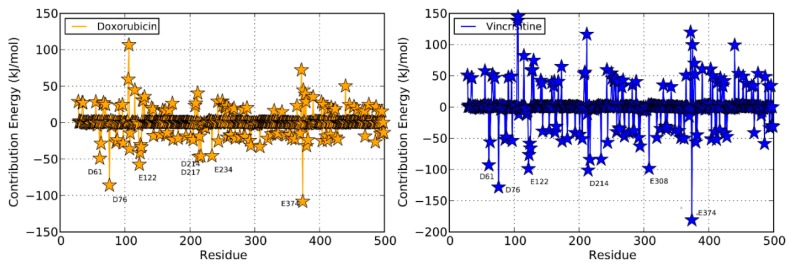
Per-residue free energy decomposition for the simulated complexes of cytarabine (**green**), daunorubicin (**red**), doxorubicin (**yellow**) and vincristine (**blue**). The most favorable interacting residues are labeled.

Figure 1 shows the docked productive binding pose of cytarabine. Three hydrogen bonds were observed, with R105, A305 and A370. The van der Waals interactions were observed with heme, R105, R212, A305, T309 and A370. The closest distance between the cytarabine and heme was 0.223 nm. As seen in Figure 2A, the RMSD of the protein-drug complex varied only about 0.05 nm from the starting structure, and similarly, the drug RMSD showed 0.05-nm differences in fluctuations throughout the MD simulations (Figure 2B). The distance between the cytarabine and heme was stable (Figure 2C), and there was no major change in the solvent accessible surface area (SASA) of cytarabine (Figure 2D). The arabinose sugar moiety of cytarabine showed higher fluctuations, whereas the amino group of the cytosine base was stable and faced towards heme throughout the simulation. Throughout the 50-ns simulation, the interacting residues with the cytarabine were R105, S119, R212, F309 and A370 (Figure S1). Moreover, there was a significant change in the orientation of the G’-helix (243–259), and due to that, the distance between the G’ helix and I helix (291–323) was increased (Movie S1). The protein movement involving phenylalanine clusters showed approximately a 0.1-nm variation from the initial structure until 20 ns; however, it was stable throughout the remaining simulation (Figure 3A). Figure 4A shows the free energy landscape (FEL) of the first two principal components using projected eigenvectors generated based on PCA. The contour map of the FEL showed seven minima in which the most populated minimum free energy cluster was extracted at 22,378 ps (Figure 4A). This structure resembled the starting conformation; however, a few interacting residues vary. Three hydrogen bonds with R212 and one hydrogen bond with A305 by cytarabine were observed in the snapshot. As observed during docking and MD simulation, the amino group of cytosine faces heme, suggesting the possible metabolic site for oxidation. The amino groups in the cytosine base, C16 and C11 in the arabinose sugar moiety of cytarabine are the first, second and third metabolic sites predicted using SMARTCyp, respectively (Figure 5A). The molecular mechanics Poisson–Boltzmann surface area (MM-PBSA) relative free energy observed for this favorable binding pose was −314.966 kJ/mol. All of the interaction energies were favorable for the chosen cytarabine complex (Table S1). The decompositions of the relative interaction energies of individual residues to cytarabine complex formation with the most favorable interactions were R105, R212, A305, I369, A370 and E374, which comprised both charged and hydrophobic amino acids (Figure 6).

#### 2.2.2. Non-Productive Binding Mode of Cytarabine Reoriented into Productive Binding Mode during MD Simulation

In another CYP3A4-cytarabine binding pose, the cytosine and arabinose moiety of cytarabine oriented parallel to the heme moiety of the CYP3A4, and the minimum distance between cytarabine and heme was 0.72 nm (Figure S2). Owing to the distance, orientation and SMARTCyp prediction, this was less likely the binding pose. Therefore, to compare this with our likely binding pose, we further performed MD simulations and found that the RMSD between protein and drug RMSD was comparatively stable (Figure S3). The distance between the heme and cytarabine was reduced around the first few nanoseconds of the MD simulations and was unstable up to 25 ns. Thereafter, the cytosine base of cytarabine reoriented itself in a metabolizable position towards the heme. As observed in favorable binding pose, it acquired stability and moved towards heme with a reduced distance of 0.4 nm at the end of MD simulations (Movie S2). The 50-ns simulation snapshot of this complex was rather similar to the productive binding pose where the amino group of the cytosine base faced against the heme of CYP3A4. This strongly suggests that the cytosine base of cytarabine may be the possible metabolic site for oxidation and proved the invaluable utility of MD simulations in deciphering protein-ligand interactions.

### 2.3. Daunorubicin

Daunorubicin is one of the anthracycline drugs used against leukemia. Daunorubicin features a tetracyclic ring structure and an amino sugar (daunosamine) [20]. Daunorubicin was reported to be metabolized by CYP3A4 in human, and the metabolic by-products are still unknown. Three major clusters were observed to have 68, 10 and 8 binding poses, respectively. We selected one productive binding pose and two non-productive binding poses. The docked results with interacting residues are shown in Table 1, and the results are discussed below. Furthermore, flexible docking was performed, and the generated poses were similar to most of the clusters obtained through rigid docking (Figure S4).

#### 2.3.1. Productive Binding Mode of Daunorubicin

In the selected productive binding pose, three hydrogen bonds were observed with E374 and R372 (Figure 1). The van der Waals interactions were observed with heme, F57, R105, R106, F108, S119, F213, F215, F304, R372 and E374. This binding pose showed that the daunosamine group faced towards heme, where the amino group was pointing outward and the methyl group faced towards heme. The tetracyclic ring structure was perpendicular to the heme. The minimum distance between the heme and daunorubicin was 0.297 nm.

In addition, these major clusters contain 68 and 70 binding poses in 1TQN and 4I3Q docking respectively. The protein backbone RMSD of this complex from the starting structure varied from 0.1 nm throughout the 50-ns simulation, while the RMSD of the drug was also stable. The distance between heme and the daunorubicin fluctuated around 0.1 nm for the 50-ns simulation (Figure 2). The SASA of daunorubicin was similar to metyrapone. Throughout the 50-ns simulation, common interacting residues with daunorubicin are R106, S119, F108, R372 and E374 (Figure S1). The side chain of R212 significantly shifted from outside to inside during the MD simulations (Movie S3). The tetracyclic ring of daunorubicin was partially stabilized via hydrophobic interactions with the phenylalanine residues F57, F106, F108 and F215. Furthermore, the tetracyclic ring of daunorubicin is positioned close to F106 and F215, where F215 formed a π stacking interaction with 0.35-nm distance. Specifically, the orientation of S119 changed during the simulation. The F–G loop (209–217) showed fluctuation around 0.15 nm up to 40 ns, but was found to be stable thereafter (Figure 3B). The FEL analysis of the representative daunorubicin complex revealed that the access to the lowest energy conformer was at the 19,876-ps snapshot, which was extracted from the most populated free energy minimum cluster out of three clusters (Figure 4B). In this conformation, R106, S119 and E374 showed hydrogen bond interactions with daunorubicin, and the tetracyclic moiety of daunorubicin was surrounded by F57 and F215. The daunosamine group of daunorubicin was accessible to heme and suggests the possibility of being the metabolic site for oxidation. The differences observed from the docked conformation were in the hydrogen bonding residues. However, other interacting residues were similar when compared to the starting conformations. SMARTCyp predicted three SOM sites in the daunosamine group (Figure 5B).

The observed MM-PBSA relative free energy for this favorable binding pose is −392.368 ± 57.719 kJ/mol (Table S1). The favorable interaction energies were van der Waals, electrostatic and solvent accessible surface area. On the other hand, polar solvation energy is unfavorable with the positive value of 210.95 ± 26.356 kJ/mol. The decomposition of the relative interaction energies of individual residues to daunorubicin complex formation with the most favorable interactions with charged amino acids were E63, D76, D174, R212, D214 and E308 (Figure 6).

#### 2.3.2. Non-Productive Binding Modes of Daunorubicin

In the first non-productive binding pose, the tetracyclic ring of daunorubicin faced towards heme, and the daunosamine group pointed outwards (Figure S4). Three hydrogen bonds were observed with T224, R372 and E374. The minimum distance between heme and daunorubicin was 0.483 nm with the AutoDock binding energy −11.69 kcal/mol. The protein backbone RMSD fluctuated around 0.15 nm from the starting structure, while the RMSD of daunorubicin was quite unchanged. The distance between the heme and drug was stable throughout the simulation (Figure S3).

In the second non-productive binding pose, daunorubicin bound 0.69 nm away from the heme in the proximal region (Figure S4). The peripheral site of daunorubicin interacts with the residue F143 and dissociated at 40 ns (Figure S3). Davydov *et al.* experimentally suggested a few important peripheral binding residues (proximal site 1–K424, F435) [21]. Since the experimentally-proven binding residues overlap with this binding pose, we wanted to evaluate the interacting behavior of this binding pose in MD simulation.

### 2.4. Doxorubicin

Doxorubicin (14-hydroxydaunorubicin) has a tetracyclic ring structure and a daunosamine group. Structurally, doxorubicin is related to daunorubicin and differs only in hydroxyl group substitution instead of hydrogen at the alkyl side. The docked doxorubicin showed two major clusters with 69 and 15 binding poses, respectively (Table 1). Out of these, one productive binding pose and one non-productive binding pose were observed. Flexible docking was also performed for this drug, and the results are shown in Figure S5.

#### 2.4.1. Productive Binding Mode of Doxorubicin

**Figure 7:**
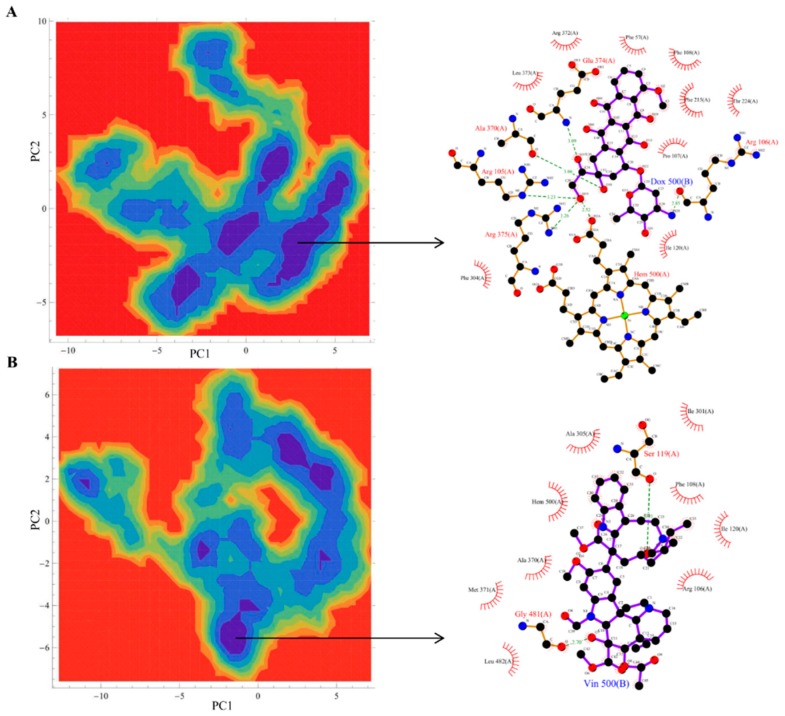
FEL and the representative lowest energy conformation of simulated drugs. The FEL of the simulated complexes based on the principal component analysis. (**A**) Doxorubicin; (**B**) vincristine. Each representative structure is displayed from the most populated free energy minimum clusters using PDBsum.

In this binding pose, five hydrogen bonds were observed with R105, S119, R372, E374 and heme (Figure 1). The minimum distance between doxorubicin and heme was 0.208 nm. The van der Waals interactions were observed in F57, D76, R105, R106, S119, F213, F215, F304, A370, R372, E374, R375 and heme. The daunosamine group of doxorubicin faced heme, and the tetracyclic ring structure is perpendicular to heme. The protein backbone RMSD was fluctuating up to 10 ns, but stable thereafter throughout the simulation (Figure 2A). Meanwhile, the drug RMSD showed no variation (Figure 2B). The distance between heme and doxorubicin was stable (Figure 2C), and the SASA of doxorubicin was lower than that of daunorubicin throughout the simulation (Figure 2D). The common interacting residues R106, S119, R372 and E374 were stable throughout the 50-ns simulation (Figure S1). F108 and A370 significantly interacted and moved closer during the simulation (Movie S4), and the other interacting residues were mostly charged and hydrophobic. The bending of the I-helix was also observed in this complex. Around 20 ns, the phenylalanine loop fluctuated <0.15 nm, but was stable further during the simulations (Figure 3A). SMARTCyp predicted three SOM sites in the daunosamine group, which is similar to the prediction results of doxorubicin (Figure 5C). The FEL analysis of the representative doxorubicin complex revealed that access to the lowest energy conformer at the 14,754-ps snapshot was extracted from the most populated free energy minimum cluster (Figure 7A). Although the doxorubicin-bound structure was similar to daunorubicin, the difference lies in the hydroxymethyl group of doxorubicin that forms several hydrogen bonds with the charged amino acids. In addition, heme formed the hydrogen bond with the hydroxymethyl group of doxorubicin. The daunosamine moiety was close to heme, suggesting it as the possible metabolic site for doxorubicin. Similar to daunorubicin, the polar solvation energy was the only unfavorable interaction energy to the doxorubicin with the final MM-PBSA relative free energy −330.171 ± 43.579 kJ/mol. All other interaction energies were favorable to this binding complex (Table S1). The decomposition of the relative interaction energies of individual residues to the doxorubicin complex formation are with the charged amino acids D61, D76, E122, D214, D217, E234 and E374 (Figure 6).

#### 2.4.2. Non-Productive Binding Mode of Doxorubicin

This binding pose showed that the 4-methoxy of the tetracycline ring projected towards heme and that the daunosamine group was outwardly oriented (Figure S5). There were two hydrogen bonds observed with E374 amino acids. The van der Waals interactions were observed with R105, R106, F108, S119, R212, F215, I301, A305, A370, R372, E374 and heme. The AutoDock binding energy was −10.24 kcal/mol, and the distance between heme and doxorubicin was about 0.275 nm. When docked with the 4I3Q structure, a similar binding pose was observed, except that the tetracycline ring was slightly tilted and there was an additional hydrogen bond interaction with P107. The protein backbone RMSD of binding pose fluctuated between 0.10–0.20 nm and was stable around 15 ns. The RMSD of the drug fluctuated from 20 ns till the end of MD simulations. The distance between heme and doxorubicin was stable throughout the simulation (Figure S3).

### 2.5. Vincristine

Vincristine is a mitotic inhibitor used in cancer therapy and a vinca alkaloid antineoplastic agent composed of two multi-ringed units: vindoline and catharanthine [9]. It is a large molecule, with a volume of 733 Å3. There are 22 different clusters in the docked vincristine that contains 10 rotatable bonds. Due to the complex structure of vincristine, there were many clusters, and it is difficult to predict the most favorable binding pose. Among the 12 poses, only one binding pose with a negative AutoDock binding energy was selected (−4.42 kcal/mol). In this binding pose, one hydrogen bond was observed with R372. This is the only binding pose with the lowest binding energy along with the SOM prediction that seems to be the most favorable binding pose for vincristine (Figure 1). In this pose, the distance between heme and vincristine was 0.263 nm. When docked with the 4I3Q crystal structure, there was a difference in hydrogen bonding residues where S119 and E374 form hydrogen bonds with the vincristine. In the 1TQN structure, there was only one binding pose that has −4.42 kcal/mol. Therefore, we subject this pose to MD simulations and found that the complex was stable (Figure 2A). Intriguingly, in flexible docking, out of 30 possible binding poses, two were in the active site, whereas all others were docked at peripheral sites (Figure S6; data were shown for the first 10 poses). The large size of the drug might be the reason for this peripheral binding.

The drug RMSD, as well as the heme-drug distance was comparatively stable (Figure 2B,C). As expected, SASA values were larger for vincristine (Figure 2D). The common interacting residues R106, S119, A370 and R372 were stable throughout the simulation (Figure S1). The side chain of R212 moved upwards and made enough space during the simulation to accommodate vincristine. This was observed in the phenylalanine loop RMSD, as well (Movie S5 and Figure 3A). R212 expanded and made enough space for vincristine binding, which was not found with any other protein-drug interaction in this study. The phenylalanine loop fluctuated around 0.3 nm, suggesting that the protein underwent conformational changes to accommodate the large vincristine molecule.

SMARTCyp predicted vindoline carbon (C54) as the primary SOM that is located near the pyrimidine ring out of two multi-ringed units, vindoline and catharanthine. The secondary metabolic site was carbon (C57) in the pyrimidine ring of the vindoline ring, and the tertiary metabolic site was carbon (C18) in the catharanthine ring of vincristine (Figure 5D). It has been reported that vincristine is metabolized into three products, namely M1, M2 and M3. The first step in vincristine metabolism is the hydroxylation of the carbon [10]. Interestingly, in our study, the same carbon was predicted to be the tertiary site of metabolism by SMARTCyp (Figure 5D). The FEL analysis of the representative vincristine complex revealed that access to the lowest energy conformer at the 23,800-ps snapshot was extracted from the most populated free energy minimum cluster (Figure 7B). The pyrimidine ring out of two multi-ringed units, vindoline and catharanthine, was accessible to heme, suggesting it as the potential metabolic site of oxidation. The final MM-PBSA relative free energy of this vincristine complex was −370.006 ± 5.057 kJ/mol (Table S1). The favorable interaction energies include van der Waals energy and electrostatic energy, *vs.* the less favorable polar solvation energy. The decompositions of the relative interaction energies of individual residues to the doxorubicin complex formation with the most favorable interactions with the residues were D61, D76, E122, D214, E308 and E374 (Figure 6).

The non-productive binding pose is shown in Figure S6. Although the binding energy was +53.43 kcal/mol, Davydov *et al.* experimentally suggested that a few important peripheral binding residues (proximal site 1–K424, F435) overlap with this binding pose; therefore, we chose it further for MD simulations [21]. The interacting residues L351, D357, N361, K424, I427, P429, Y432, P434, F435, G436, S437, M445, R446, L449 and K453 were observed in this binding pose. The frequently-interacting peripheral vincristine binding residues are R109, S110 and S113. Due to its comparatively large size, it was not stable and dissociated after 10 ns in MD simulations (Figure S3).

### 2.6. Control Docking and Simulation

The control docking was performed using another crystal structure of CYP3A4 (4I3Q) to evaluate the binding pattern that gave a similar drug orientation to the active site (Figures S2, S4–S6). The docking results of both structures were similar except a few differences observed in the hydrogen bonding. The crystal structure selected for our study, *i.e.*, 1TQN, does not have co-complex ligands. Therefore, we have used the crystal structure of BEC (3UA1) bound to CYP3A4 for comparison (Figure S7). Irrespective of a few limitations, AutoDock has the ability to predict the binding modes with minimum RMSD difference when compared to the co-crystallized drug.

To validate the non-productive binding pose, we used the unfavorable AutoDock binding mode of metyrapone, which is bound a 0.4-nm distance from the heme. During the simulation, the binding position moved closer to heme and re-oriented itself towards the crystal structure conformations by the end of the simulation (Movie S6). This suggested MD simulations of small molecular size ligands with a non-productive binding mode corrected to the favorable binding mode in 50-ns simulations. Contrastingly, for large ligands, the accelerated MD simulation techniques are required to achieve the reorientation. To assess the SMARTCyp server, we used cyclophosphamide, warfarin and bromoergocryptine (BEC), which are the substrates of CYP3A4 (Figure S8). The productive binding mode and SOM of cyclophosphamide have been predicted with remarkable accuracy. Similarly, the warfarin was docked as a productive binding mode, and SOM prediction is in line with a previous report [22]. This drug is metabolized by CYP3A4 into 10-hydroxywarfarin through the hydroxylation process. The SMARTCyp server rightly predicted the tertiary metabolizing site where the hydroxyl group replaces hydrogen at the 10th position of the warfarin in the oxidative degradation process (Figure S8). The productive binding mode predicted by AutoDock is in good agreement with the experimentally-validated structures (Figure S7). BEC contains two moieties: (1) cyclic peptide; and (2) the lysergic acid subunit. *In vivo* metabolite analysis has revealed that BEC is oxidized by CYP3A4 at the cyclic peptide moiety, with the 8′-mono- and 8′,9′-di-hydroxy derivatives being the major products [23]. However, SMARTCyp predicted the metabolic site at the lysergic acid moiety, which is in contrast to the experimental data. Hence, for the validation of SMARTCyp, we selected 54 substrates of CYP3A4 in which their metabolic products are known [24]. Out of those substrates, SMARTCyp correctly predicted the SOM for 46 substrates (Table S4). The reason for a few false predictions by SMARTCyp is possibly due to the limitations explained by Rydberg *et al.* [25]. In addition, there may be minor metabolites that have not been experimentally proven. This suggested that the SMARTCyp server is capable of predicting the substrate metabolic site reasonably well.

### 2.7. Discussion

CYPs are anchored to the membrane by an N-terminal transmembrane α-helix, and there is evidence that the globular domain dips into the membrane [26]. Human CYPs are connected to the membrane via an N-terminal α-helix, and this helix is replaced by hydrophilic residues to facilitate crystallization. However, because of the difficulties of dealing with membrane-bound CYPs, most biophysical experiments and simulations have been performed with soluble CYPs [6]. In our study, we also used the soluble form of CYPs without the lipid layer. Our primary aim was to find the binding orientation of the anticancer drugs cytarabine, doxorubicin, daunorubicin and vincristine with CYP3A4. We performed rigid and flexible docking approaches, both of which gave similar orientations of the drugs.

Recently, the crystallographic structure of CYP3A4 in complex with BEC, an agonist for dopamine receptor, has been resolved [27]. It indicates that the CYP3A4-BEC complex is oriented in a productive mode, where the primary sites of oxidation are closest to the heme [23]. The BEC is next to R212, which shifts aside to allow access to the heme. Similarly, another crystal structure complex CYP3A4-ritonavir (HIV protease inhibitor) involves R212 and forms the hydrogen bonding. The crystal structure of the CYP3A4-ritonavir complex shows that ritonavir is anchored to the active site via hydrogen bond with S119 [28]. The computational study on CYP3A4 by Park *et al.* suggested that S119 is an important active site residue that could stabilize the inhibitor and substrate binding via hydrogen bond formation [29]. Shahrokh *et al.* used quantum mechanics calculations and MD simulations to probe the reactivity of 4-hydroxy-tamoxifen, a primary metabolite of the anticancer drug tamoxifen, for dehydrogenation and to generate representative CYP3A4 and drug configurations [30]. This approach helped to identify the 4-hydroxy-tamoxifen binding modes consistent with the *in vitro* metabolism. Since the R212A mutant produced half of the dehydrogenated product as wild-type, R212 was proposed to be a key residue that affects 4-hydroxy-tamoxifen dehydrogenation by imposing both steric and electrostatic inhibition of the rebound process, but only when it is oriented towards the active site.

Likewise, in the case of cytarabine, R212 serves as an important active site residue that stabilizes cytarabine via hydrogen bonding. In the case of cytarabine, the important and repeated amino acids are R212, R105, R372, A370 and L483. The SMARTCyp predicted the primary SOM as the amino group in the cytosine moiety of cytarabine (Figure 5A). MD simulations of cytarabine with a non-productive binding mode reoriented itself to the favorable binding mode, suggesting the cytosine moiety of cytarabine as the preferable SOM. It appears that cytarabine may be deaminated by the removal of the amino functional group as ammonia, and the ketone group replaces the amino group. The secondary SOM site was at the methoxy group of arabinose sugar moiety, and this possibly results in the demethylation reaction, resulting in a net loss of one carbon and two hydrogen atoms. However, the docking and MD simulations do not support the feasibility of secondary SOM. In addition, the next SOM site suggests the possible dehydrogenation of drug, where R212 was the key residue that may execute this reaction.

Analysis of the crystal structure CYP3A4 revealed that the antifungal drug ketoconazole is hydrogen bonded to R372, and there was a π-stacking interaction with F304 [31]. Similarly, the tetracyclic moiety of daunorubicin formed a π-stacking interaction with F215. Further, the X-ray model of the CYP3A4-erythromycin complex showed that drug bound to CYP3A4 in a non-productive orientation [31]. There were no specific polar interactions between the erythromycin molecule and the protein, but the close proximity of four phenylalanine side chains suggested that the complex was partially stabilized via hydrophobic interactions. In addition, the association of ritonavir-like molecules was also driven by heme coordination, whereas hydrophobic interactions provided by the side groups define the binding mode.

Both daunorubicin and doxorubicin binding modes and interactions were similar. Daunorubicin and doxorubicin showed that the S119 and R372 formed the important interactions with CYP3A4. The experimental metyrapone results showed that S119 was involved in ligand binding. The metyrapone co-complex crystal structure revealed essentially no conformational changes in the protein [32]. SMARTCyp predicted that both of the drugs have similar primary and secondary SOMs in the daunosamine moieties. As far as daunorubicin and doxorubicin were concerned, the predicted SOM, as well as the docked binding mode suggest that the daunosamine group is the possible metabolism site of drugs. The favorable productive binding poses have the primary, secondary and tertiary SOMs oriented towards the heme for these respective drugs (Figure 5B,C). It appears that the docking and SMARTCyp predictions suggest that daunorubicin and doxorubicin may be involved in the deamination reaction by the removal of the amino functional group as ammonia, and the ketone group replaces the amino group.

Probably, due to large size of vincristine, there were many clusters in the docking. Only one binding pose had the favorable AutoDock binding energy. The hydrogen bond in vincristine was with the R372 amino acid, and many other hydrophobic interactions were also observed. It is to be noted here that the phenylalanine loop has specific fluctuations (Figure 3A). This fragment serves as a specific site to interact with the limited number of substrate molecules [6]. Interestingly, SMARTCyp predicted that primary and secondary metabolic sites faced heme in the favorable binding mode for vincristine, while the predicted tertiary site had already been proven by an experimental study [10]. PCA suggests that unlike other protein-drug interactions in the study, R212 expands and makes enough space for vincristine binding. This was similar to the crystal structure of the CYP3A4-erythromycin complex where conformational rearrangements take place in the F–G connecting loop to accommodate the drug [20]. Moreover, our analysis demonstrated that certain residues have favorable drug-specific interactions, which is supported by the findings of Hayes *et al*. [24].

There is more experimental evidence for the existence of a peripheral ligand binding in CYP3A4 (progesterone), as well as for CYP2C8 [21]. However, a recent study has confronted the progesterone peripheral binding site and suggested that it might be due to the crystallographic artifact [27]. Sevrioukova *et al.* summarized the substrate binding to CYP3A4 as follows. (i) Substrates are not locked in the CYP3A4 active site and may dissociate and rebind during different stages of the catalytic cycle; (ii) Before moving into the active site cavity, the ligand may saturate the peripheral binding site of CYP3A4, and once saturated, it moves into the active site [6]. Since the experimentally-proven residues were the interacting residues in the docked complexes, we were interested to know whether this may be an appropriate route for compounds that moved directly from the peripheral site into the active site. However, the MD simulations suggested that the drugs unbound at different intervals, which is in line with previous observations (Figure S3). Perhaps it might be possible that the drugs rebind during different stages from which drug molecules could be transported to the active site for metabolism. The MM-PBSA analysis supported the dominance of hydrophobic forces in the CYP3A4-drug association, which is well known [6,24]. The calculated relative free energy was energetically favorable for all of the drug complexes. Additionally, the prediction accuracy of SMARTCyp has been validated by a dataset comprising 54 small molecules with their established metabolic profiles. The prediction of the SOM of these molecules is given in Table S4, which highlights the potential accuracy of this web server (SOMs of 46/54 were predicted correctly).

Computational methods have been widely used to investigate important features of CYPs with the aim to predict positions of oxidation and to determine important structural features that confer selectivity. Several existing structures of CYPs have been useful to further improve our knowledge about the underlying ligand recognition mechanism of CYPs. In our study, one of the limitations was that the CYP3A4-drug complexes may get trapped in local minima and never get out of those in the 50-ns simulations for larger molecules. We argue that the smaller ligands (cytarabine and metyrapone) with a non-productive binding mode are corrected to the favorable binding mode in 50-ns simulation (Movies S2 and S6), which is in line with a previous study involving valproic acid [11]. However, there were no drastic changes observed in the 50-ns simulation for the bigger drugs, like daunorubicin, doxorubicin and vincristine. This may be due to the large-sized ligand and the MD time scale limitations.

## 3. Experimental Section

### 3.1. Docking

Human CYP3A4 crystal structures were retrieved from the Protein Data Bank (IDs: 1TQN [33] and 4I3Q) [34]. The missing residues in the structures were built using UCSF MODELLER; the modeled structures were energy minimized in order to remove steric clashes, and the lowest energy structure was selected. Cytarabine, daunorubicin, doxorubicin and vincristine drugs, which are clinically used in combinatorial cancer therapy, were selected from the National Cancer Institute Database [35]. These drugs are proven substrates for CYP3A4 (http://www.drugbank.ca/). The three-dimensional structures of these drugs, as well as control drugs were converted and optimized using Open Babel software (http://openbabel.org/). Ligand structures were energy minimized using the steepest descent and conjugate gradient method. Docking was performed with AutoDock 4.2, and AutoDockTools was used to prepare, run and analyze the docking results [36]. Kollman charges, solvation parameters and polar hydrogens were added to the protein structure, and the Gasteiger charge was assigned to the ligands. The grid box size was set at 82 × 82 × 82 Å in the catalytic region of the heme moiety with 0.375 grid spacing. The Lamarckian genetic algorithm was used to search the best conformers with a hundred docking runs of each ligand. The MOE program was used for the flexible docking procedure with its default parameters.

### 3.2. Molecular Dynamic Simulations

All simulations were carried out using the Gromacs 4.6 package [37] with the CHARMM27 force field [38]. The protonation states of histidines were assigned (residue 27 HID, other histidines HIE). Drug parameters were prepared using the SwissParam web server [39]. The drug parameters are provided in the figshare link [40]. Steepest descent minimization followed by a conjugate gradient minimization was applied to the solvated system. A cubic box with periodic boundary conditions was set up. The systems were then solvated using the simple point charge explicit water model and were neutralized by adding an appropriate number of counter ions. During the equilibration dynamics, the system was coupled to the Berendsen thermostat to maintain a temperature of 300 K and to the Berendsen barostat to maintain a pressure of 1 bar. A cut-off of 1.4 nm was used for the computation of short-range non-bonded interactions, and the particle mesh Ewald (PME) method was used for computing long-range electrostatic interactions. All bonds were constrained using the LINear Constraint Solver (LINCS) algorithm, allowing an integration time step of 2 fs. The MD runs were carried out in the NPT ensemble (50 ns) for each system.

Images were generated using the UCSF Chimera package and PyMOL Molecular Graphics System, Version 1.7.4, Schrödinger, LLC. Graphs were drawn using Matplotlib. To calculate relative free energy, g_mmpbsa [41], which uses the molecular mechanics Poisson–Boltzmann surface area (MM-PBSA) sourced from the Gromacs and APBS packages [42], was adopted. All computational studies were performed on a Dell PowerEdge server with the CentOS6 GNU/Linux operating system.

### 3.3. Heme Parameters

We used the available CHARMM27 force field in Gromacs 4.6. The missing hydrogen database was added to heme in the file aminoacids.hdb (Table S2) and was adopted from an earlier study [43]. A copy of the CHARMM27 force field file aminoacids.hdb is available at figshare [44] along with the details. The bond between the Fe atom of heme and the SG atom (CYS residue 442) was detected using chainsep id. Fe- and S-related missing parameters were added manually in the topology of the CHARMM27 force field and are provided in Table S3.

### 3.4. Principal Component Analysis and Free Energy Landscape Analysis

The high-amplitude concerted motion in the protein trajectories through the eigenvectors of the covariance matrix of protein atomic fluctuations can be unveiled using PCA [45]. The first 20 projection eigenvectors of the protein were extracted from simulated drug complexes and analyzed for their cosine content in which the first two eigenvectors (PC1 and PC2) having cosine content less than 0.2 were used to define the FEL. We applied the g_sham module of the Gromacs package to calculate FEL. The contour maps of the FEL were generated using the trial version of Mathematica.

## 4. Conclusions

In conclusion, this study presents the probable binding modes of anticancer drugs that extend the understanding of CYP3A4-drug interaction. Ultimately, we are able to expose that multiple simulations for different binding modes for the same drug are likely to provide the correct binding mode for small-sized drug molecules. The MM-PBSA analysis supports the dominance of hydrophobic forces in the CYP3A4-drug association, which is well known. This is a unique study to understand the binding efficiencies of these drugs and highlights the potential undermining aspects in drug designing. By combining multiple approaches, we can design drugs with less side effects that are highly specific, efficient and easily excretable from the body. This study may also be useful to design analogs with reduced toxicity and to provide valuable insights into the metabolism of the cancer drugs.

## Supplementary Materials

Supplementary materials can be accessed at: http://www.mdpi.com/1420-3049/20/08/14915/s1.
