# Supplementary material for: A Combined Molecular Docking/Dynamics Approach to Probe the Binding Mode of Cancer Drugs with Cytochrome P450 3A4
Source: Molecules. 2015 Aug 14;20(8):14915–35. doi: 10.3390/molecules200814915 (PMC6332164; doi:10.3390/molecules200814915)
Supplement: Supplementary file 1 [file molecules-20-14915-s001.zip › molecules-20-14915.pdf]

Supplementary Information

Table S1. Productive and non-productive poses relative free energies between CYP34A and the drugs.

Drug	Van der Waals Energy (kJ/mol)	Electrostatic Energy (kJ/mol)	Polar Solvation Energy (kJ/mol)	SASA Energy (kJ/mol)	SAV Energy (kJ/mol)	Binding Energy (kJ/mol)
Cytarabine (Productive)	−121.622 ± 9.772	−81.448 ± 33.392	−4.982 ± 8.353	−13.25 ± 0.909	−144.428 ± 5.859	−314.966 ± 29.680
Cytarabine (Non-productive)	−138.810 ± 7.523	−155.362 ± 37.929	100.326 ± 13.811	−12.973 ± 0.795	−146.693 ± 6.006	−303.077 ± 27.082
Metyrapone	−127.696 ± 11.585	−68.375 ± 22.550	62.406 ± 8.800	−13.193 ± 0.756	−149.259 ± 6.102	−248.015 ± 23.611
Daunorubicin I (Productive)	−203.434 ± 17.374	−208.843 ± 83.064	210.95 ± 26.356	−22.943 ± 1.359	−258.4 ± 10.700	−392.368 ± 57.719
Daunorubicin II (Productive)	−276.988 ± 10.448	−33.372 ± 44.375	214.774 ± 26.182	−37.502 ± 3.240	−290.006 ± 37.502	330.254 ± 49.875
Daunorubicin (Non-productive)	−111.542 ± 5.301	−53.111 ± 48.901	80.374 ± 4.813	−13.187 ± 2.510	−175.499 ± 8.388	−175.120 ± 18.863
Doxorubicin I (Productive)	−238.466 ± 13.318	−145.484 ± 46.220	254.051 ± 15.636	−25.446 ± 0.741	−266.874 ± 11.095	−330.171 ± 43.579
Doxorubicin II (Non-productive)	−265.343 ± 12.590	−58.674 ± 48.883	212.670 ± 12.360	−24.995 ± 0.773	−269.597 ± 11.071	−316.816 ± 36.495
Vincristine I (Productive)	−314.776 ± 18.427	−3.752 ± 63.442	217.104 ± 34.320	−33.187 ± 1.348	−352.517 ± 15.064	−370.006 ± 65.057
Vincristine II (Non-productive)	28.810 ± 10.321	−325.712 ± 79.822	16.697 ± 5.127	−4.288 ± 3.411	−60.082 ± 12.454	−285.283 ± 32.817

Table S2. Hydrogen database for heme. We retrieved the available CHARMM27 force field in Gromacs 4.6, and the missing hydrogens were added to heme (red color) in the file aminoacids.hdb. The file is provided in the link http://figshare.com/articles/Cytochrome_CHARMM_heme_parameter_file/1254117.

```
!       O2A   O1A             O2D  O1D
!         \\ //                 \\ //
!          CGA                   CGD
!           |                     |
!    HBA1--CBA--HBA2  HA    HBD1--CBD--HBD2
!           |          |          |
!    HAA1--CAA-HAA2  _CHA_ HAD1--CAD--HAD2
!           |       /     \       |
!          C2A---C1A       C4D---C3D
!           |     |         |     |
!HMA1\      |     |         |     |      /HMD1
!HMA2-CMA--C3A    NA       ND    C2D--CMD-HMD2
!HMA3/       \   / \       / \   /       \HMD3
!             C4A   \     /   C1D
!            /       \   /       \
!      HB--CHB        FE         CHD--HD
!            \       /   \       /
!             C1B   /     \   C4C        HAC
!HMB1\       /   \ /       \ /   \       /
!HMB2-CMB--C2B    NB       NC    C3C--CAC
!HMB3/      |     |         |     |     \\  /HBC1
!           |     |         |     |      CBC
!          C3B---C4B       C1C---C2C        \HBC2
!           |       \_CHC_/       |
!          CAB         |         CMC--HMC3
!         //  \        HC       /  |
!        CBB  HAB          HMC1  HMC2
!       /   \
!    HBB1  HBB2
```

HEME 16

1	1	HA	CHA	C1A	C4D
1	1	HB	CHB	C4A	C1B
1	1	HC	CHC	C1C	C4B
1	1	HD	CHD	C1D	C4C
3	4	HMA	CMA	C3A	C2A
2	6	HAA	CAA	C2A	CBA
2	6	HBA	CBA	CAA	CGA
3	4	HMB	CMB	C2B	C1B
1	1	HAB	CAB	C3B	CBB
2	3	HBB	CBB	CAB	C3B
3	4	HMC	CMC	C2C	C1C
1	1	HAC	CAC	CBC	C3C
2	3	HBC	CBC	CAC	C3C
3	4	HMD	CMD	C2D	C1D
2	6	HAD	CAD	C2D	CBD
2	6	HBD	CBD	CAD	CGD

Table S3. Parameters for Fe-S bond, angles, and dihedral values. The covalent bond between iron (Fe) of heme and sulfur (S) was detected using chainsep id in the pdb2gmx module of Gromacs. We used Fe- and S related missing parameters (from CHARMM27) into the topology file appropriately where that bond/angle/dihedral is defined for Fe-S bond.

[bondtypes]							
; i	j	func	b0	kb			
SG	FE	1	0.232	209200.0			
[angletypes]							
; i	j	k	func	th0	cth	ub0	cub
CT2	SG	FE	5	100.6	418.4	0.0	0.0
SG	FE	NPH	5	90.0	836.8	0.0	0.0
SG	FE	NPH	5	90.0	836.8	0.0	0.0
SG	FE	NPH	5	90.0	836.8	0.0	0.0
SG	FE	NPH	5	90.0	836.8	0.0	0.0
; ###							
X	CS	SS	X	9	0.20	0.0	3
CA	CB	SG	FE	9	0.20	0.0	3
HB1	CB	SG	FE	9	0.20	0.0	3
HB1	CB	SG	FE	9	0.20	0.0	3
; ###							
X	FE	SS	X	9	0.00	0.0	4
CB	SG	FE	NPH	9	0.00	0.0	4
CB	SG	FE	NPH	9	0.00	0.0	4
CB	SG	FE	NPH	9	0.00	0.0	4

Table S4. The known substrates of CYP3A4 were used for validating SMARTCyp server.

S. No.	Name of the Substrate	SmartCYP Prediction
1	Erythromycin	Predicted correctly
2	Alprazolam	Predicted correctly
3	Midazolam	Predicted correctly
4	Sertraline	Predicted correctly
5	Citalopram	Predicted correctly
6	Amitriptyline	Predicted correctly
7	Mirtazapine	Predicted correctly
8	Trazodone	Predicted correctly
9	Nefazodone	Wrong prediction
10	Donepezil	Predicted correctly
11	Ziprasidone	Predicted correctly
12	Clozapine	Predicted correctly
13	Zolpidem	Predicted correctly
14	Eszopiclone	Predicted correctly
15	Zaleplon	Predicted correctly
16	Carbamazepine	Predicted correctly
17	Zonisamide	Predicted correctly
18	Buspirone	Predicted correctly
19	Ifosfamide	Predicted correctly
20	Imatinib	Predicted correctly
21	Clarithromycin	Predicted correctly
22	Ritonavir	Predicted correctly
23	Delavirdine	Predicted correctly
24	Nevirapine	Predicted correctly
25	Atorvastatin	Wrong prediction
26	Cerivastatin	Wrong prediction
27	Simvastatin	Wrong prediction
28	Verapamil	Predicted correctly
29	Diltiazem	Predicted correctly
30	Guanfacine	Wrong prediction
31	Disopyramide	Predicted correctly
32	Amiodarone	Predicted correctly
33	Bosentan	Predicted correctly
34	Propranolol	Predicted correctly
35	Finasteride	Wrong prediction
36	Letrozole	Predicted correctly
37	Toremifene	Predicted correctly
38	Flutamide	Predicted correctly
39	Oxybutynin	Predicted correctly

Table S4. *Cont.*

S. No.	Name of the Substrate	SmartCYP Prediction
40	Tolterodine	Predicted correctly
41	Cyclobenzaprine	Predicted correctly
42	Omeprazole	Predicted correctly
43	Zileuton	Predicted correctly
44	Montelukast	Predicted correctly
45	Astemizole	Wrong prediction
46	Cocaine	Predicted correctly
47	Caffeine	Predicted correctly
48	Dronabinol	Predicted correctly
49	Domperidone	Predicted correctly
50	Dapsone	Predicted correctly
51	Dextromethorphan	Predicted correctly
52	Pioglitazone	Predicted correctly
53	Nateglinide	Predicted correctly
54	Saxagliptin	Wrong prediction

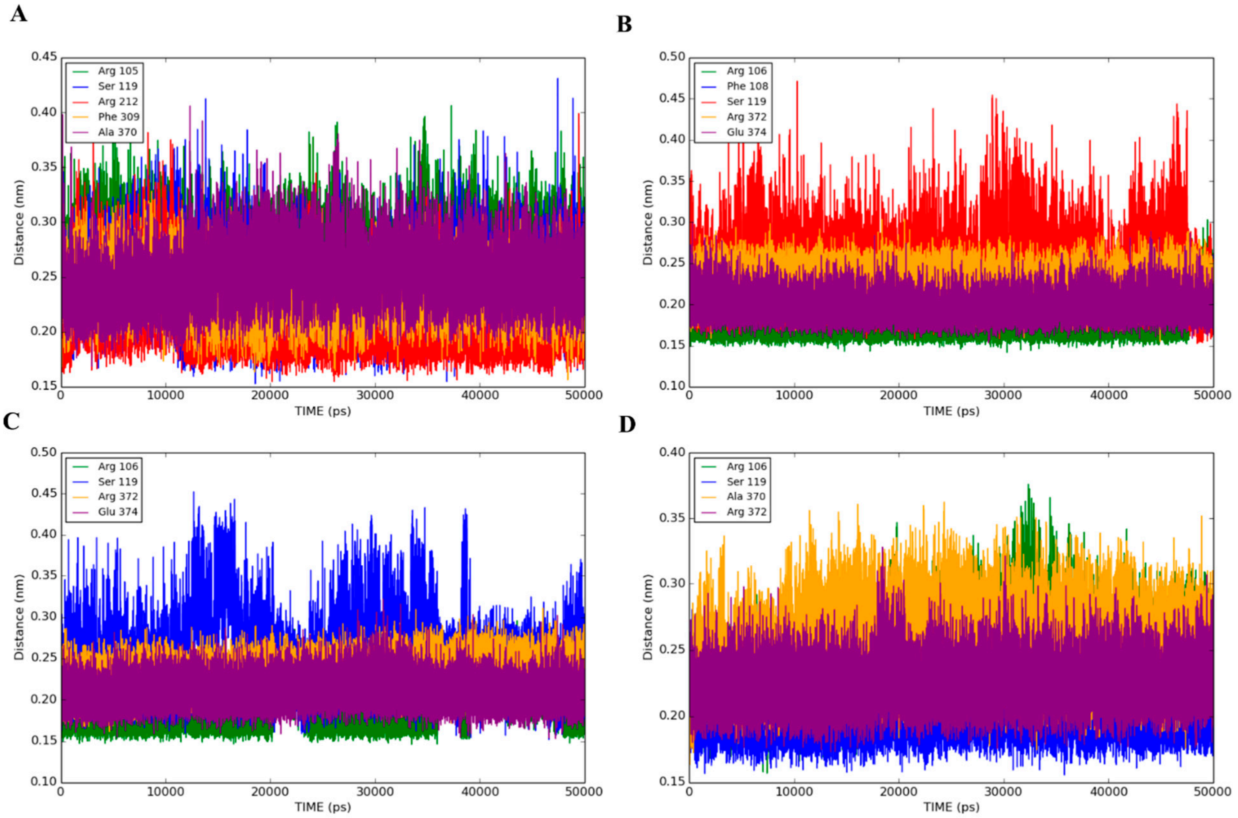

Figure S1. The distance between drug and nearby amino acids in the productive binding poses. (**A**) Cytarabine (**B**) Daunorubicin (**C**) Doxorubicin (**D**) Vincristine. Amino acids are labeled accordingly as given in the figures of the complexes.

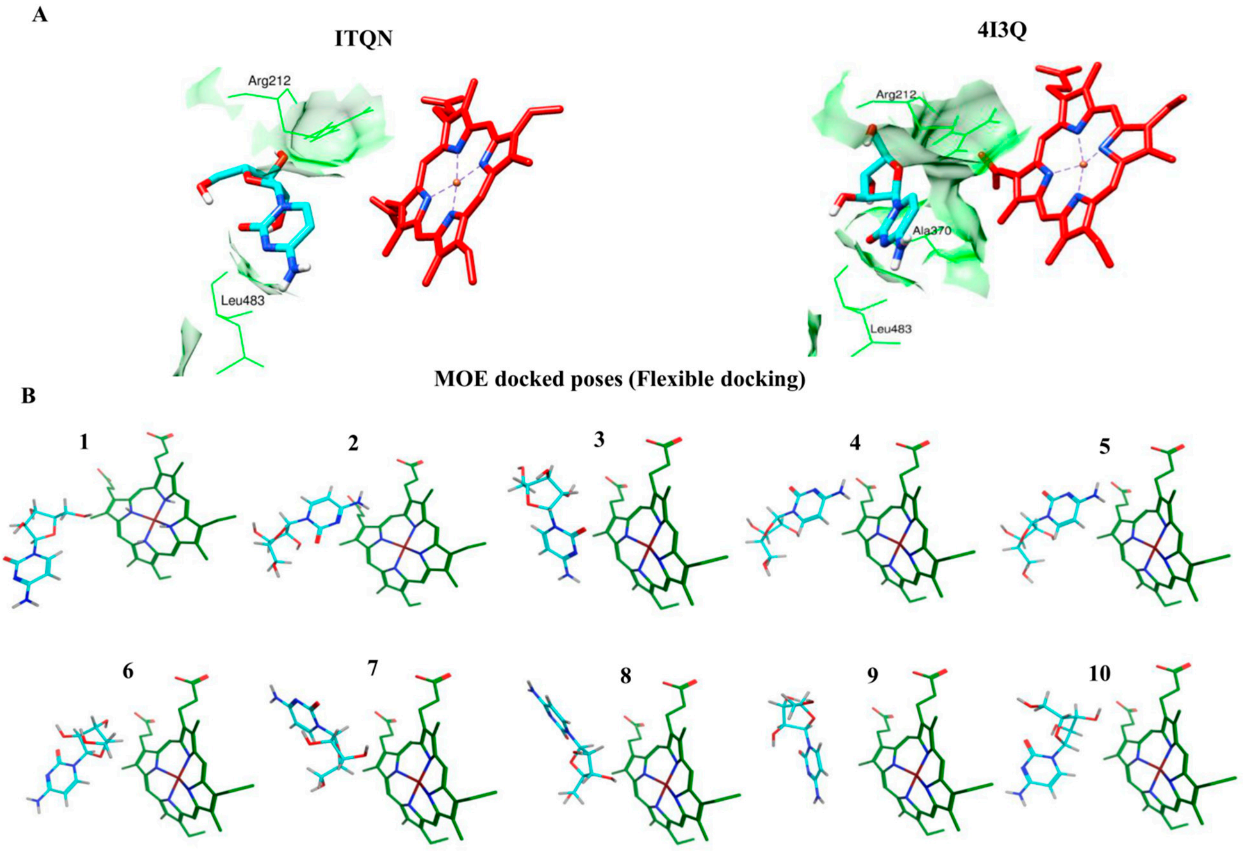

Figure S2. The molecular docking poses of cytarabine. (**A**) Non-productive binding mode of cytarabine with CYP3A4 (PDB ID|1TQN and 4I3Q) obtained through rigid docking. Only the heme (red color in stick representation), ligand (cyan color in stick representation), and hydrogen bonding residues (green color in line representation) are shown. A few hydrogen bonding residues are not shown for clarity; (**B**) Flexible docking poses of cytarabine.

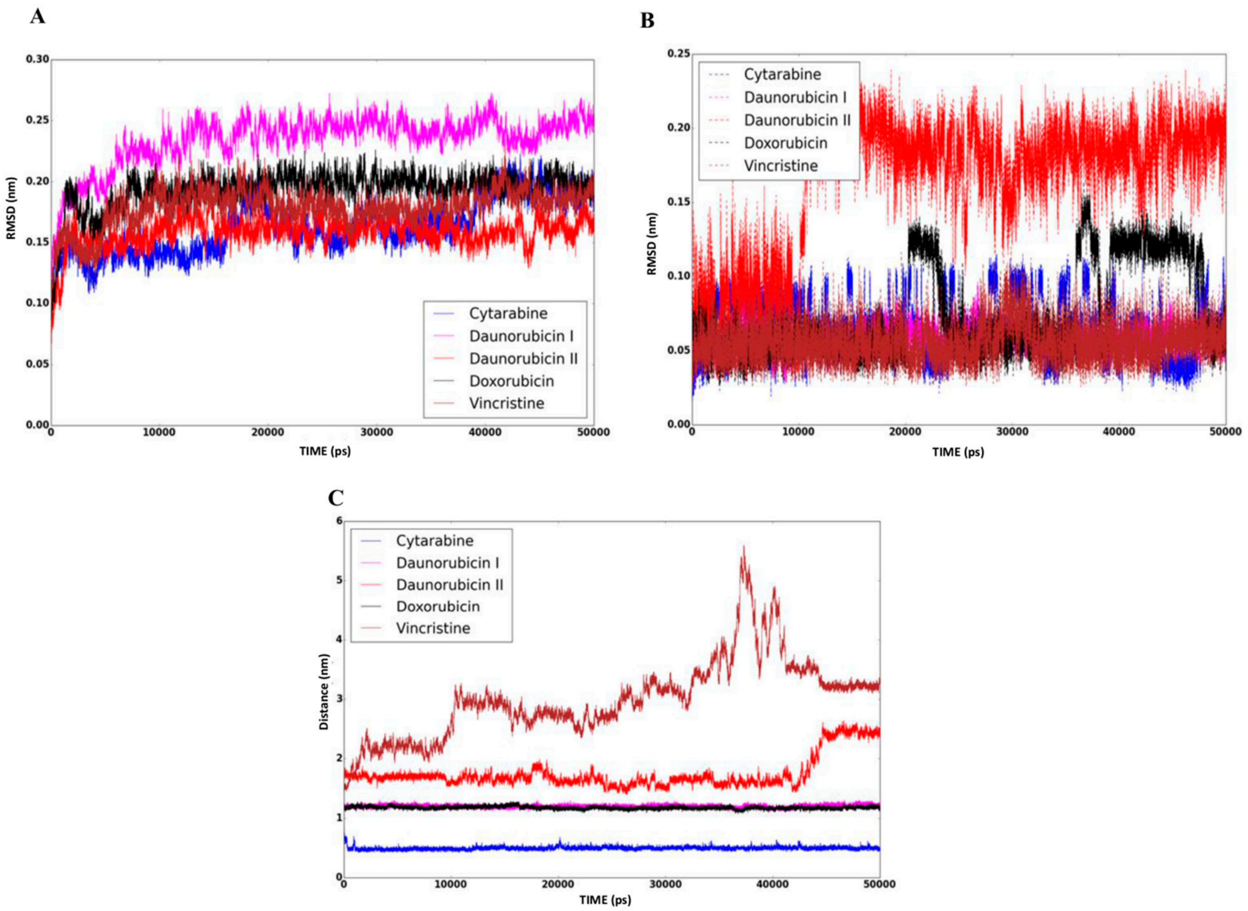

Figure S3. Analysis of MD simulations. Figures were drawn using Matplotlib. Non-productive binding pose of cytarabine (blue), daunorubicin I (pink), daunorubicin II (red), doxorubicin (black), and vincristine (brown). (**A**) Root mean square deviation (RMSD) of the protein backbone atoms, with respect to the initial structure for 50 ns simulations; (**B**) Ligand RMSD, with respect to the initial structure for 50 ns simulations; (**C**) Heme-drug distances were measured throughout the 50 ns simulations. Since the heme-drug distances were calculated using centers of mass of two groups (g_dist), they differ from the heme-drug distances provided in Table 1 that used the minimum distance between two groups.

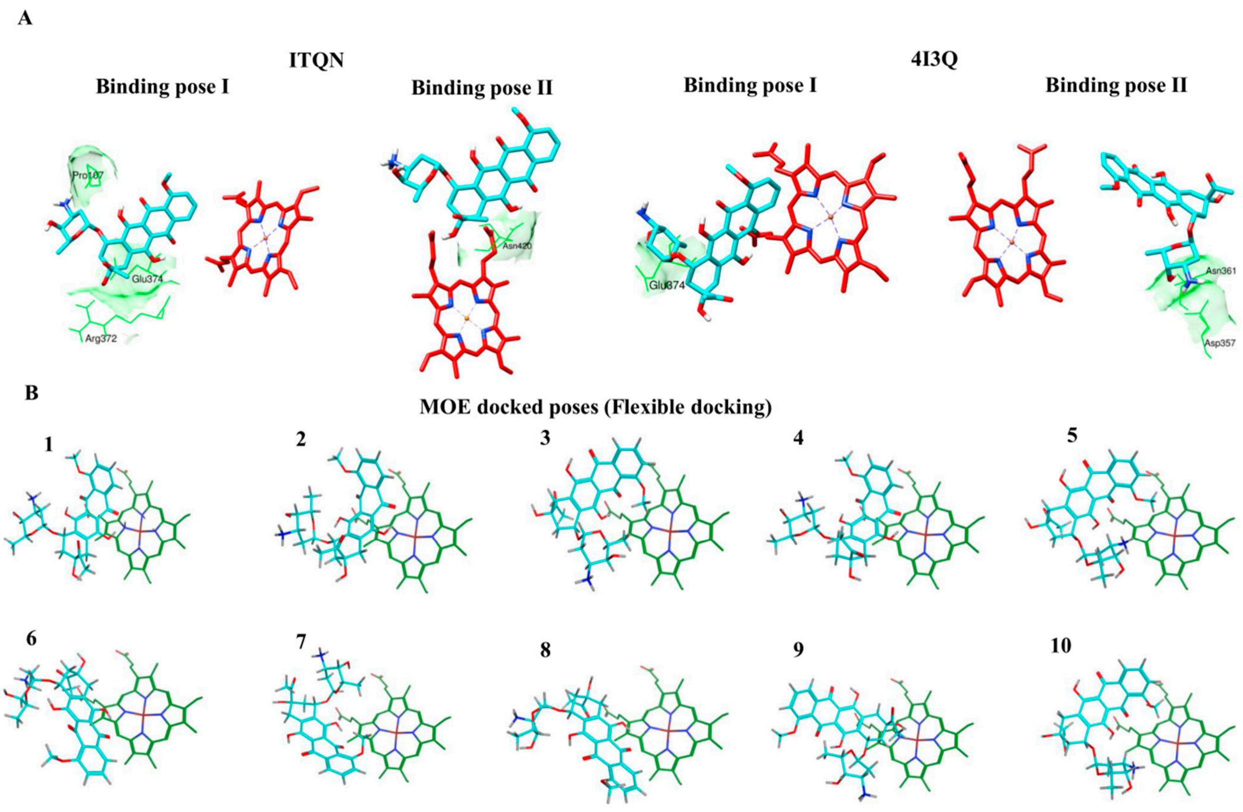

Figure S4. The molecular docking poses of daunorubicin. (**A**) Non-productive binding modes of daunorubicin with CYP3A4 (PDB ID|1TQN and 4I3Q) obtained through rigid docking. Only the heme (red color in stick representation), ligand (cyan color in stick representation), and hydrogen bonding residues (green color in line representation) are shown. A few hydrogen bonding residues are not shown for clarity; (**B**) Flexible docking poses of daunorubicin.

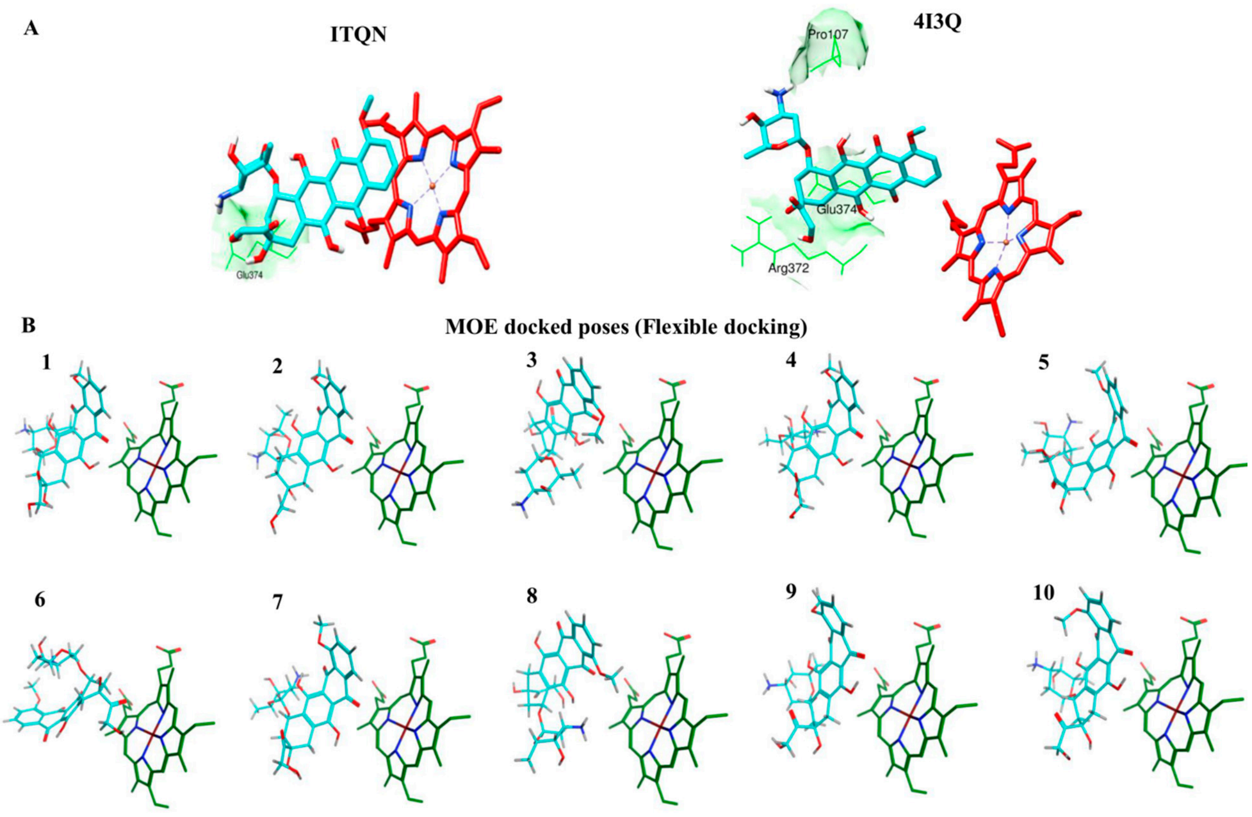

Figure S5. The molecular docking poses of doxorubicin. (**A**) Non-productive binding mode of doxorubicin with CYP3A4 (PDB ID|1TQN and 4I3Q) obtained through rigid docking. Only the heme (red color in stick representation), ligand (cyan color in stick representation), and hydrogen bonding residues (green color in line representation) are shown; (**B**) Flexible docking poses of doxorubicin.

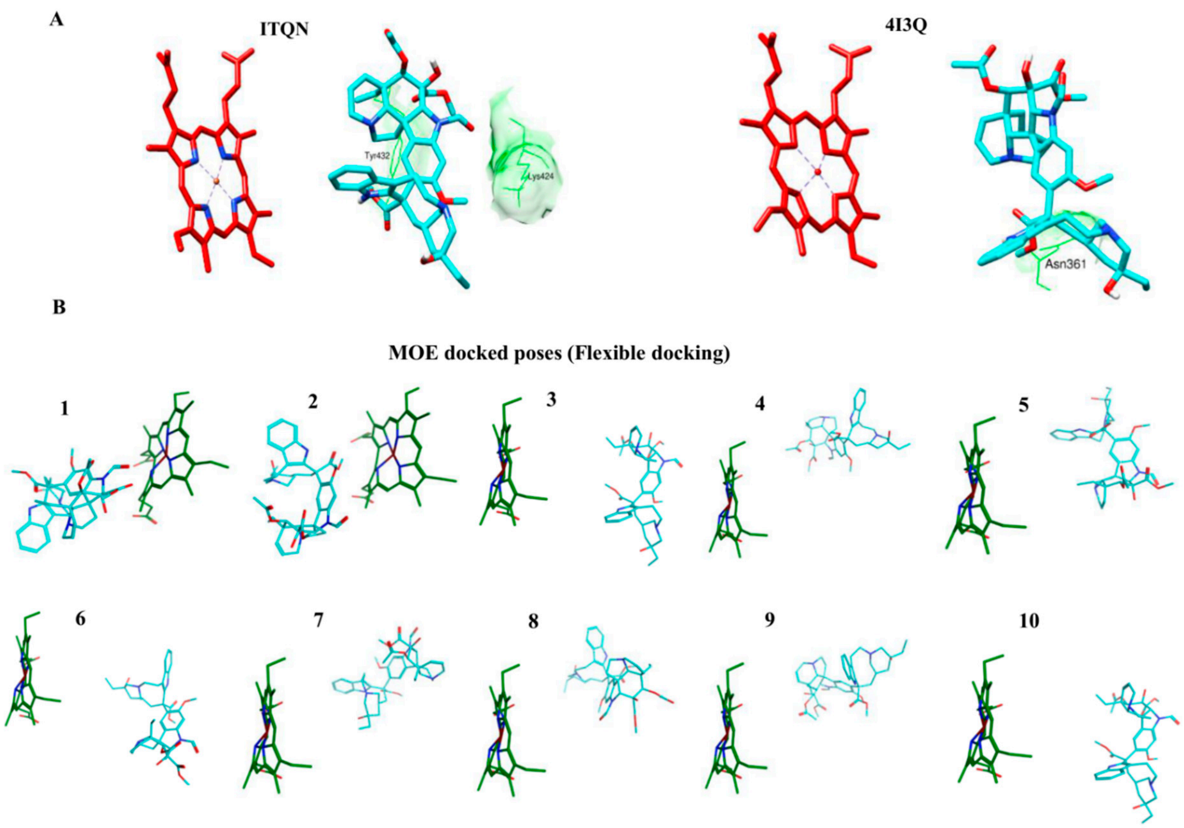

Figure S6. The molecular docking poses of vincristine. (**A**) Binding modes of vincristine with CYP3A4 (PDB ID|1TQN and 4I3Q) obtained through rigid docking. Only the heme (red color in stick representation), ligand (cyan color in stick representation), and hydrogen bonding residues (green color in line representation) are shown. A few hydrogen bonding residues are not shown for clarity; (**B**) Flexible docking poses of vincristine.

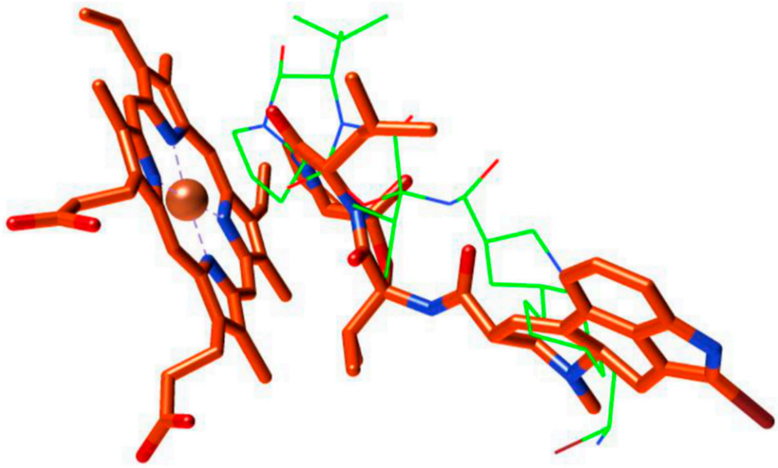

Figure S7. Binding pose generated by AutoDock with reference to the crystallized structure of bromoergocryptine (BEC). Since there is no co-crystal complex for 1TQN, we have used the crystal structure of BEC (3UA1) bound to CYP3A4 for comparison. Heme is shown in red stick representation. The docked drug-binding pose is shown as lines, and the crystal structure-binding pose is shown as stick. The productive binding mode of BEC and the experimental pose are compared.

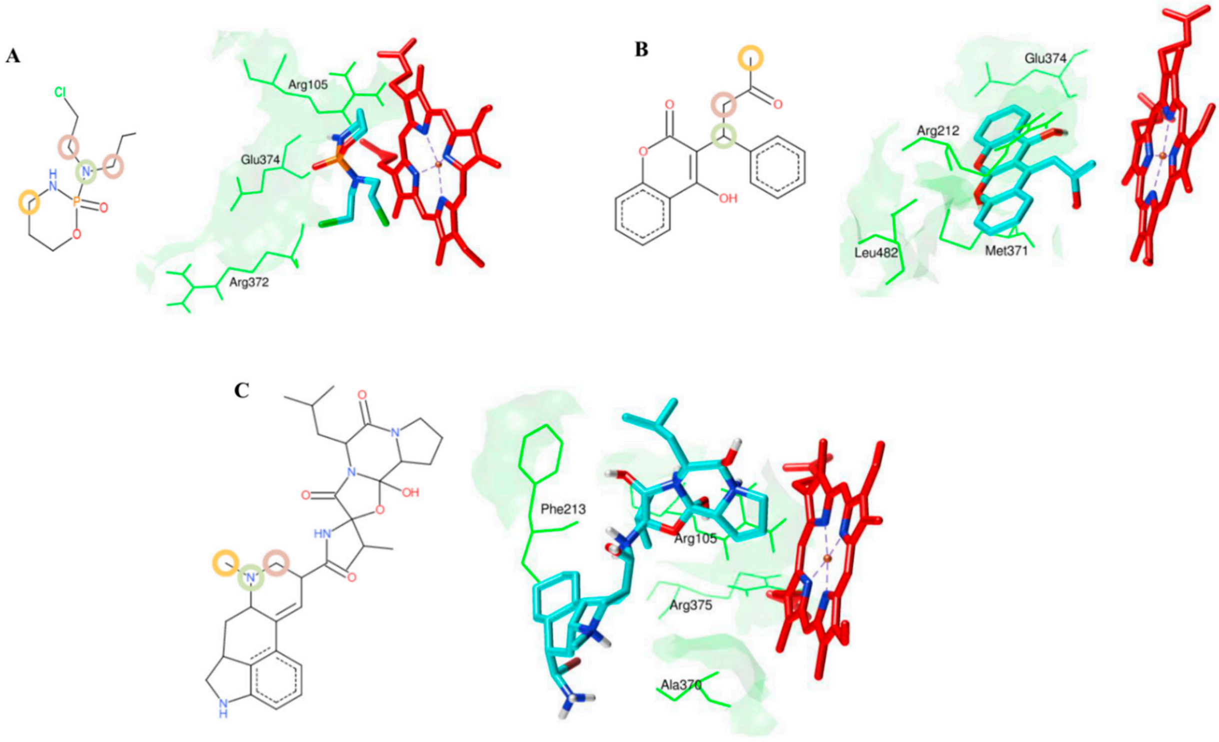

Figure S8. The prediction accuracy of SMARTCyp and the productive docking poses of control. (**A**) Cyclophosphamide metabolizing sites were correctly predicted by SMARTCyp, where the metabolites are 4-hydroxycyclophosphamide and dechloroethyl cyclophosphamide. One of the productive binding modes of cyclophosphamide is shown here; (**B**) SMARTCyp server predicted correctly the tertiary metabolizing site where hydroxyl group replaces hydrogen at the 10th position of warfarin along with the productive binding mode of warfarin; (**C**) A previous *in vivo* metabolite analysis has revealed that bromoergocryptine (BEC) is oxidized by CYP3A4 at the cyclic peptide moiety, with the 8′-mono- and 8′,9′-dihydroxy derivatives being the major products. However, SMARTCyp predicted the metabolic site at the lysergic acid moiety. The productive binding mode of BEC is shown along with the interacting residues.

Movie S1. The movie show the conformational changes of domains along the simulation trajectory in the CYP3A4-cytarabine complexes. Protein is shown in ribbon representation and functionally important residues are shown in stick representation. The movie was created using PyMOL Molecular Graphics System, Version 1.7.4 Schrödinger, LLC.

Movie S2. The movie for 50 ns run of the non-productive CYP3A4-cytarabine complex. Protein is shown in ribbon representation. Heme (red) and cytarabine (cyan) are shown in stick representation. As seen in the movie, non-productive binding mode of cytarabine orients itself towards the productive mode at the end of simulation. The movie was created using UCSF chimera with 50 ns of the whole trajectory.

Movie S3. The movie shows conformational changes of the domains along the simulation trajectory in the CYP3A4-daunorubicin complexes. The side chain of R212 significantly migrated from outside to inside during the simulation**.** Protein is shown in ribbon representation and functionally important residues are shown in stick representation. The movie was created using PyMOL Molecular Graphics System, Version 1.7.4 Schrödinger, LLC.

Movie S4. The movie shows the conformational changes of the domains along the simulation trajectory in the CYP3A4-doxorubicin complexes. F108 and A370 played an important role in the interaction and moved closer during the simulation. Protein is shown in ribbon representation and functionally important residues are shown in stick representation. The movie was created using PyMOL Molecular Graphics System, Version 1.7.4 Schrödinger, LLC.

Movie S5. The movie shows conformational changes of the domains along the simulation trajectory in the CYP3A4-vincristine complexes. The side chain of R212 moved upward and made enough space during the simulation to accommodate vincristine. Protein is shown in ribbon representation and functionally important residues are shown in stick representation. The movies were created using PyMOL Molecular Graphics System, Version 1.7.4 Schrödinger, LLC.

Movie S6. The movie for 50 ns run of the unfavorable CYP3A4-metyrapone complex (Control). Protein is shown in ribbon representation. Heme (red) and cytarabine (cyan) are shown in stick representation. During the simulation, the binding mode of metyrapone moves closer to heme and re-oriented itself towards the crystal structure conformations by the end of MD simulations. The movie was created using UCSF chimera with 50 ns of the whole trajectory.
